# Two initial vaccinations with the Bm86-based Gavac^plus ^vaccine against *Rhipicephalus (Boophilus) microplus *induce similar reproductive suppression to three initial vaccinations under production conditions

**DOI:** 10.1186/1746-6148-6-43

**Published:** 2010-09-16

**Authors:** Milagros Vargas, Carlos Montero, Dunia Sánchez, Danny Pérez, Mario Valdés, Aymé Alfonso, Marisdania Joglar, Héctor Machado, Elsa Rodríguez, Luis Méndez, Ricardo Lleonart, Marisela Suárez, Erlinda Fernández, Mario P Estrada, Alina Rodríguez-Mallón, Omar Farnós

**Affiliations:** 1 Clinical Trials Department, Center for Genetic Engineering and Biotechnology, 31th Avenue and 190, Havana, 10600, Cuba; 2 Parasitology Department, National Center for Parasitology, Avenue San Antonio-Rincón, Km 1 1/2, Havana, Cuba; 3 Animal Health Department, Center for Genetic Engineering and Biotechnology, 31th Avenue and 190, Havana, 10600, Cuba; 4 Animal Biotechnology Division, Center for Genetic Engineering and Biotechnology, 31th Avenue and 190, Havana, 10600, Cuba

## Abstract

**Background:**

The cattle tick, *Rhipicephalus *(*Boophilus*) *microplus*, affects livestock production in many regions of the world. Up to now, the widespread use of chemical acaricides has led to the selection of acaricide-resistant ticks and to environmental contamination. Gavac^plus ^is a subunit vaccine based on the recombinant Bm86 tick antigen expressed in yeast, capable to control infestations of *R. microplus *under controlled and production conditions. The vaccine constitutes the core element of broad control programs against this ectoparasite, in which acquired immunity in cattle to Bm86 is combined with a rational use of acaricides. At present, the conventional vaccine scheme consists of three doses that should be administered at weeks 0, 4 and 7, followed by a booster every six months.

**Results:**

In this study we assayed a reduction in the number of the initial doses of Gavac^plus^, evaluated the time course and the level of bovine anti-Bm86 antibodies elicited, and analyzed the vaccine effect on ticks engorging on immunized cattle under production conditions. Following three different immunization schemes, the bovines developed a strong and specific immune response characterized by elevated anti-Bm86 IgG titers. A reduction in the weight of engorging female ticks, in the weight of the eggs laid and also in *R. microplus *viable eggs percentage was obtained by using only two doses of Gavac^plus ^administered at weeks 0 and 4, followed by a booster six months later. This reduction did not differ from the results obtained on ticks engorging on cattle immunized at weeks 0, 4 and 7. It was also demonstrated that anti-Bm86 antibody titers over 1:640, measured in bovines immunized at weeks 0 and 4, were sufficient to affect weight and reproductive potential of female ticks as compared with ticks engorging on unvaccinated animals. In addition, no statistically significant differences were detected in the average weight of eggs laid by ticks engorged on immunized cattle that showed anti-Bm86 specific titers in the range of 1:640 to 1:81920.

**Conclusion:**

The administration of two initial doses of Gavac^plus ^containing 100 μg of Bm86 antigen to non-immunized cattle under production conditions is sufficient to affect the weight and the reproductive capacity of *R. microplus *engorging females. According to these results, cattle herds' manipulation and vaccine costs could be potentially reduced with a positive impact on the implementation of integrated control programs against *R. microplus*.

## Background

*Rhipicephalus microplus *is an ectoparasite that currently affects the cattle industry in many regions of the world and it is also an important vector for the transmission of parasites in diseases such as anaplasmosis and babesiosis [1,2].

The use of acaricides is the most extended prophylactic and therapeutic method to control ectoparasites. However, some relevant drawbacks regarding their use are the development of acaricide-resistant ticks after repeated treatments, the chemical contamination of cattle-derived products and of the environment [3-5]. In the last years, these factors led to the selection of alternative strategies aiming to achieve a better control of ectoparasites under safer approaches [6-8].

Efforts were primarily focused in the identification and characterization of concealed Bm86-like antigens as vaccine candidates [9-13]. Other tick antigens such as BmTI, serine protease inhibitors and 4D8 have been described in recent years with marked potential for the development of novel or combined vaccines [14-17]. However, only two Bm86-based vaccines commercially available have been used in the field in different countries involving the immunization and monitoring of a large number of bovines [18-21]. Nowadays, it is known that using Bm86 for cattle immunization turns into a highly effective control method if it is used as part of an integrated control program in which acaricides are simultaneously applied according to the infestation index detected [21,22]. The most remarkable benefits regarding the use of Bm86-derived vaccines are the reduction in reproductive capacity of engorging females and in the frequency of acaricide treatments.

The implementation of such programs using Gavac^plus ^implies an immunization scheme that starts with the administration of three doses at weeks 0, 4, and 7, followed by boosters every six months [22-24]. This method showed its effectiveness in the induction of high antibody titers in bovines in spite of their race, sex, or reproductive category [25]. However, this regimen demands an arduous manipulation of cattle herds in the first two months since treatment is performed. Therefore, the implementation of a different schedule that allows a reduction of manipulation and vaccine costs constitutes a desirable fact for both practical and commercial points of view. The Bm86 antigen contained in Gavac^plus ^is obtained in a highly particulated form, with high homogeneity and reproducibility in *P. pastoris *yeast [26], under strict controls of a well-recognized biotechnology industry [8,27]. Taking into consideration that this antigen is immunogenically superior with regard to the monomeric form of the protein [28], this fact prompted us to investigate the capacity of two doses of the vaccine to elicit immune responses and tick damage similar to those achieved with the use of three inoculations. In this work, we demonstrated that the generation of specific antibodies and the physical damage caused to female ticks engorging on cattle immunized under production conditions remained invariable after reducing in one dose the number of initial administrations of the Gavac^plus ^vaccine.

## Methods

### Immunization experiments in cattle under production conditions

Ten active production farms, located in the same geographical area (22°41'N, 82°53'W) in Candelaria municipality, Pinar del Rio Province (on the Western part of Cuba), were selected for the experiment. These farms are located approximately 0.85 Km separated from each other within an area of about 36 Km^2 ^and have equivalent conditions of humidity, temperature, grass type and indexes from moderate to high of natural infestations with *R. microplus*. Farms were randomly assigned to each experimental group, and different immunization schemes with the Bm86-based Gavac^plus ^vaccine were assayed. After immunizations took place, a sample of 30% of bovines per farm was selected at random to measure the biological parameters of ticks under engorgement as it will be described below. In all cases, cross-bred (*Bos taurus × Bos indicus*) non-immunized bovines, with approximately the same age and composition, were used. Manipulation of cattle herds during the immunization trials was similar to all groups and was carried out following the standard procedures for experimentation with animals. The trials and the distribution of animals by farms were done in the following way: Animals in Farm 1 (86 animals), Farm 4 (102 animals) and Farm 7 (192 animals) were immunized at weeks 0, 4 and 7; animals in Farm 2 (106 animals), Farm 6 (161 animals) and Farm 9 (150 animals) were immunized at weeks 0 and 4; animals in Farm 3 (116 animals), Farm 5 (151 animals) and Farm 8 (168 animals) were vaccinated at weeks 0 and 7, while Farm 10 (131 animals) was considered as a control group with unvaccinated animals. Animals were immunized by using a deep intramuscular injection, 21 × 11/2" needles and 2 mL of Gavac^plus^, containing 100 μg of recombinant Bm86 antigen emulsified in the oil-based adjuvant Montanide 888. Additional booster doses were given to all vaccinated animals six months after they were first immunized.

### Serum collection and assessment of anti-Bm86 antibody titers

Blood samples were taken from randomly selected bovines at weeks 0, 4, 7, 12, 16, 20, 24 and 27, in order to study the time course and anti-Bm86 specific antibody levels. These animals (30% of bovines per farm) were also considered for measuring the biological parameters of ticks under engorgement. Serum samples were stored at -20°C until use. Anti-Bm86 IgG titers were determined by ELISA as it was previously described [29], with minor modifications. Titers from individual animals were expressed as the maximum dilution having an OD_492nm _higher than two folds the average OD from a bovine seronegative control serum.

### Determination of the average weight and reproductive capacity of ticks engorging on vaccinated animals

As the experiment was conducted under production conditions, adult female ticks engorging on vaccinated cattle were collected from a representative number of animals at approximately each time point in which serum samples were also taken (subsequently from weeks 0 to 27). The effects produced by the three schemes of immunization with Gavac^plus ^were evaluated by measuring the following parameters: the average weight of engorging females, the average weight of eggs laid per gram of engorging female (referred as normalized weight of eggs laid that expresses the reproductive capacity of females considering that the eggs laid weight is directly related to the weight of the engorging female), and then the percentage of viable eggs.

For these objectives, adult engorging females were washed with distilled water, dried, and individually weighted. They were immobilized on adhesive tapes and placed into acclimatized chambers at 28°C, 90% of humidity and photoperiods of 12 hours, until oviposition. The number of eggs laid from every single tick was then weighted and loaded in glass flasks with cotton caps. They were kept in similar conditions of temperature and humidity until hatching. The percentage of viable eggs (hatchability) was determined by direct counting of larvae and eggs from each flask sample. The comparison of hatchability between the two time points selected (before starting the immunization trials and after finishing them) was conducted for the three schemes evaluated.

### Statistical analysis

Specific anti-Bm86 antibody titers were determined from a representative number of immunized animals at each farm. Geometric mean titers (GMT) and standard deviations were calculated for all the experimental groups. Differences in anti-Bm86 GMT, in the weight of engorging females, and in normalized weight of eggs laid in each immunized group were determined from weeks 0 to 27 using the non-parametric Friedman's test. These two last parameters measured on ticks when the experiment ended were compared with values obtained prior to immunization using a Mann-Whitney's test, with *p *< 0.05. The medians of the percentages of viable eggs from ticks engorging on bovines prior to immunization and after vaccination were compared for each scheme by a Kruskal Wallis and a Dunn's test. The statistical software GraphPad Prism v4.0 was used in all cases.

## Results and Discussion

### Different immunization regimens with Gavac^plus ^induce a potent response of anti-Bm86 specific antibodies

**Figure 1:**
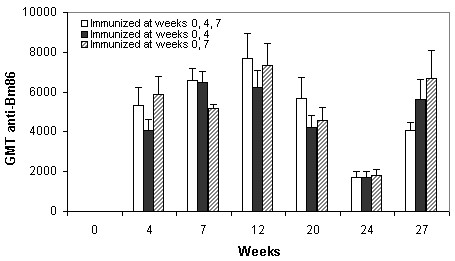
**Geometric mean of antibody titers against Bm86 determined by ELISA**. At week 0 specific antibodies to Bm86 were not detected. A Friedman's test was employed at each time point for comparison of GMT. No statistical differences were found when the three immunization schemes were compared. Standard deviation bars in the positive sense are indicated.

Previous to the first immunization, serum samples from cattle at each farm were assayed by ELISA for anti-Bm86 antibodies and were found seronegative. Following the three immunization schemes using the Bm86-based Gavac^plus ^vaccine (at weeks 0, 4, 7; 0, 4 and 0, 7), all the experimental animals developed a strong and specific humoral immune response characterized by high anti-Bm86 IgG titers. As it was proved by measurements undertaken until week 27, specific antibody titers were in all cases over 1:640, persisted in time and, as it was expected, notably increased after revaccination at week 24 (Figure 1). The immunogenicity found in these schemes was in accordance with results reported during controlled and field immunization trials using Bm-like antigens, where elevated titers were promptly detected [30,6,21,31,22]. At the present time, it has been demonstrated that certain amounts of antibodies against Bm86 correlate with damage on ticks engorging on immunized cattle [21,32]. Previous experiments also suggested that 1:640 constitutes an approximate titer cut-off where a damaging effect mediated by specific antibodies can be expected on ticks to some extent [21]. In cattle vaccinated with Bm86, titers decrease over time as the antigen remains naturally hidden to the bovine immune system during tick infestations occurrence. Therefore, re-immunizations must be practiced for maintenance of circulating antibodies. In this case, no statistically significant differences (*p *> 0.05) in the geometric mean of titers were found when the animals immunized with different schemes were compared along the entire experiment. As it can be seen in Figure 1, the reduction in one dose of Gavac^plus ^did not affect the humoral immune response generated. These antibody levels contrast with recent results obtained by other authors [33], who reported low anti-Bm86 titers after three immunizations with Gavac^plus ^in a trial to assess the efficacy of Bm86 antigens from an African strain of *B. microplus *and from *B. annulatus *(Israeli strain). Despite that fact, these authors found the highest efficacy by using Gavac^plus^, thus confirming the existence of the epitopes needed to induce damage against various tick species and different *B. microplus *strains. The discrepancy they noticed between efficacy and low titers could be attributed to the different proteins used for coating the plates in the ELISA for quantification of anti-Bm86 antibodies.

The level of the immune response obtained with two initial vaccinations of Gavac^plus ^suggested that an effective action could be expected on ticks. Comparisons on the regimens of vaccination and their effects on ticks were conducted in this investigation under standard production conditions. One farm remained with unvaccinated animals and was considered as a negative control.

### Two initial doses of Gavac^plus ^affected the reproductive potential of ticks engorging on immunized cattle

**Figure 2:**
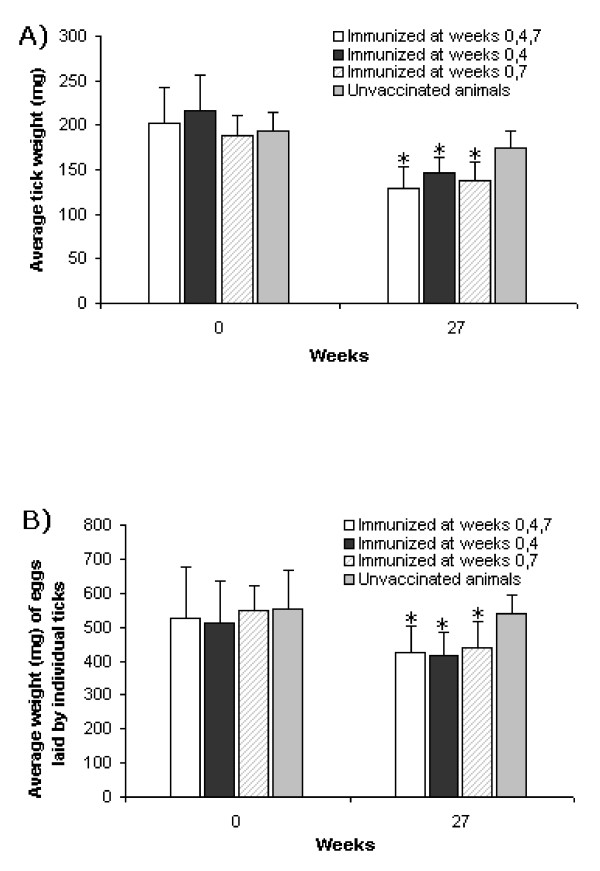
**Average values of tick weight (A) and weight of eggs laid by an individual tick (B) measured at the beginning and at week 27 of the experiment**. After vaccination, these variables showed a statistically significant reduction in the three schemes assayed (indicated with an asterisk at week 27), according to a Mann-Whitney's test with *p *< 0.05. Mean values measured from ticks engorged on unvaccinated animals did not show a significant variation. Standard deviation bars in the positive sense are indicated.

**Figure 3:**
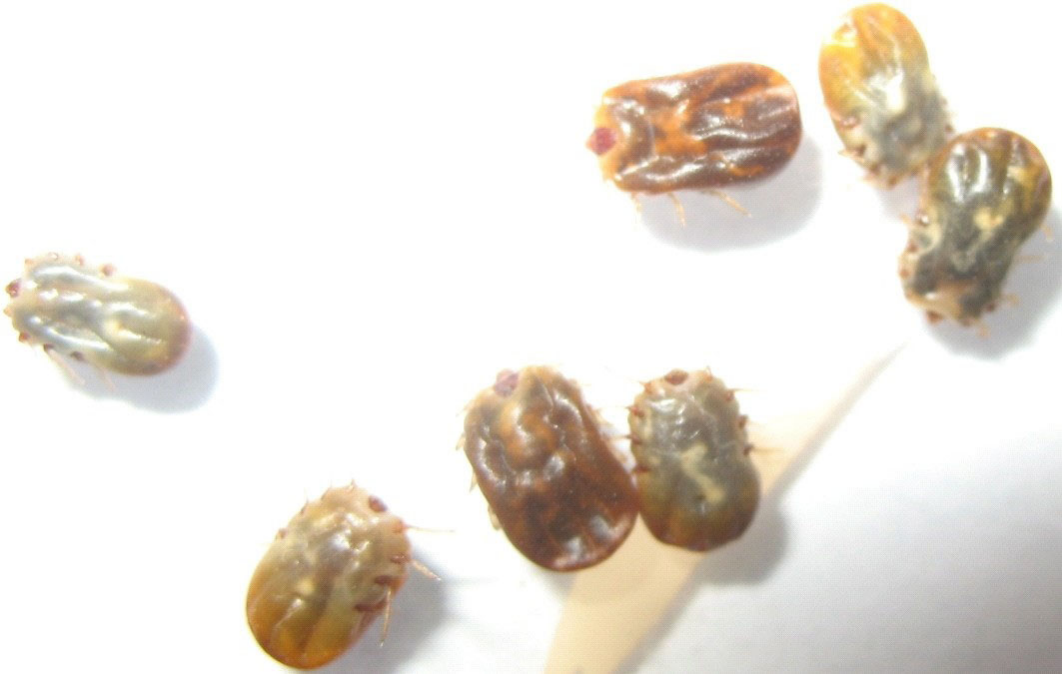
**Ticks detached from cattle immunized at weeks 0 and 4 were visibly damaged showing a characteristic dark-red/yellow color and a dehydrated appearance**.

The efficacy of this type of anti-tick vaccine, applied to cattle under production conditions, has been mostly assessed by measuring the reduction in the weight of female ticks and the impact on their reproductive capacity [6,21,23,22]. Therefore, the effect of the three immunization regimens with Gavac^plus ^was investigated by measuring a possible reduction on the average tick weight, on the average weight of the eggs laid by an individual tick and on the total percentage of viable eggs. These measurements were conducted in a sample of bovines at each farm, from weeks 0 to 27. In the immunized groups, the weight of engorging females was significantly reduced after vaccination (Mann-Whitney's test, *p *< 0.05) compared with values at the beginning of the experiment (Figure 2A). No significant variation was detected in ticks detached from unvaccinated animals in the evaluated period. The analysis of the normalized weight of eggs laid also showed lower mean values at the end of the assay with the three implemented schemes (Mann-Whitney's test, *p *< 0.05) (Figure 2B). Most importantly, no significant differences were found in the weight of females engorging on immunized animals (Table 1) and in the normalized weight of eggs laid (Table 2) when the three schemes were compared at each time point using a Friedman's test (*p *> 0.05). On the other hand, ticks engorged on vaccinated cattle were also visibly damaged (Figure 3), showing a characteristic dark-red/yellow color previously described in immunization experiments with Bm86-based vaccines [13]. As previously stated, a reduction of the reproductive capacity of ticks engorged on immunized cattle tends to promote reduction of eggs and larvae in surrounding ground, which in consequence diminishes the population of ticks and delays the necessity for acaricide treatments. The latter fact reduces as well the selective pressure related to the generation of strains with acquired resistance to chemicals. Despite the "knock-down" effect is not a feature of this kind of vaccine, the reduction in the frequency of application of acaricides is one of the most valuable effects of vaccination that can be expected at long-term [6,8].

**Table 1:** Average weight of engorging female ticks detached from immunized cattle.

Average weight (in mg) of engorging females [95% confidence interval]			
**Week**	**Immunizations at 0, 4, 7**	**Immunizations at 0, 4**	**Immunizations at 0, 7**

0	202 [183-220]^a^	215 [197-229]^a^	189 [167-201]^a^
4	178 [127-224]^a^	186 [125 -285]^a^	146 [121-152]^a^
7	169 [141-192]^a^	170 [157-196]^a^	150 [138-166]^a^
10	161 [152-173]^a^	175 [163-192]^a^	179 [161-194]^a^
16	174 [170-191]^a^	176 [136-202]^a^	187 [168-201]^a^
20	171 [156-187]^a^	181 [171-188]^a^	175 [160-192]^a^
27	129 [117-141]^a^	145 [141-157]^a^	138 [126-144]^a^

Identical letters within the same row indicate that no statistically significant differences were found when the three schemes were compared. A Friedman's test was employed.

**Table 2:** Mean values of normalized weight of eggs laid from ticks engorged on vaccinated animals.

Mean weight (in mg) of eggs laid by individual ticks [confidence interval 95%]			
**Week**	**Immunizations at 0, 4, 7**	**Immunizations at 0, 4**	**Immunizations at 0, 7**

0 4	525 [385-613]^a^ 438 [375-498]^a^	512 [477-585]^a^ 388 [275-683]^a^	548 [512-604]^a^ 426 [345-501]^a^
7	456 [413-503]^a^	493 [432-556]^a^	475 [415-542]^a^
10	421 [385-443]^a^	435 [384-485]^a^	463 [448-509]^a^
16	480 [464-509]^a^	489 [371-576]^a^	498 [472-523]^a^
20	448 [401-470]^a^	442 [374-459]^a^	449 [395-512]^a^
27	425 [372-475]^a^	415 [375-491]^a^	440 [396-479]^a^

Identical letters within the same row indicate that no statistically significant differences were found when the three schemes were compared. A Friedman test was used for comparison.

In this experiment, the hatchability (percentage of viable eggs) showed a moderate (although statistically significant) reduction by the end of the experiment in two of the three immunization schemes assayed with respect to values observed before vaccination (Table 3). Comparison through the non-parametric Kruskal-Wallis and the Dunn's tests (*p *< 0.05) indicated that immunizing at weeks 0 and 7 had no evident impact on the percentage of hatched eggs, which differed with the effect of immunizations performed at weeks 0, 4, 7 and 0, 4. Although the other analyzed parameters did not vary within the three regimens proposed, this result suggested a superior vaccine performance with the 0, 4, 7 and the 0, 4 schemes.

**Table 3:** Percentage of eggs that hatched determined from ticks engorged on vaccinated cattle before and after immunizations.

	Medians of hatchability (%)		
Immunization scheme	0, 4, 7	0, 4	0, 7

Before immunization	96.77	96.10	94.89

After immunization	83.16	83.14	90.71*

Reduction (%)	13.61	12.96	4.18*

*Indicates a statistically significant difference (*p *< 0.05) with respect to the groups immunized following the 0, 4, 7 and the 0, 4 schemes.

### Anti-Bm86 antibody titers ranging from 1:640 to 1:81920 induced similar effects on the reproductive capacity of ticks following immunization of cattle at weeks 0 and 4

**Figure 4:**
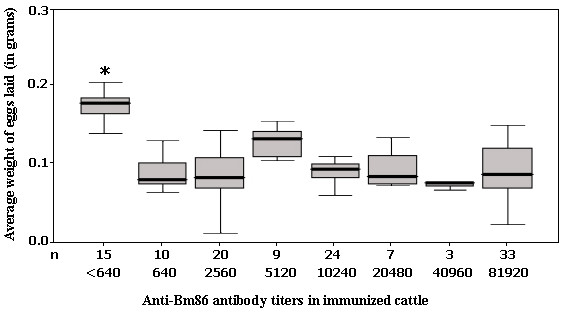
**Average weight of eggs laid by ticks engorged on groups of cattle immunized at weeks 0 and 4, showing anti-Bm86 antibody titers ranging from 1:640 to 1:81920**. Animals included in the comparison were randomly selected from vaccinated and unvaccinated animals. The mean weight of eggs laid (black lines), confidence interval for 95% (grey squares) and standard deviation bars (in both senses) are indicated. A statistically significant difference (Friedman's test, indicated with an asterisk) was found between the negative control group and vaccinated animals. n: indicates the number of bovines that were grouped by antibody titers as indicated in the abscise axis.

To evaluate the relationship between the anti-Bm86 antibody titers and the effect of their variability in the reproductive potential of engorging females after immunization at weeks 0 and 4, the weight of eggs laid was compared using ticks detached from bovines with substantial differences in the levels of specific antibodies to Bm86. We considered the widest possible range, and bovines were divided into seven groups according to titers that ranged from 1:640 to 1:81920. Animals from the farm that were no vaccinated were taken into account as an additional group for the analysis. The comparison showed that IgG titers over 1:640, reached after immunizations at weeks 0 and 4, were sufficient to affect the reproductive potential of female ticks. The mean weight value of eggs laid by ticks engorged in the groups of animals with titers in the range of 1:640 to 1:81920, statistically differed (*p *< 0.05) from those values measured on ticks engorged on unvaccinated animals. Interestingly, we found no significant differences in this parameter once the immunized cattle was able to exhibit specific titers against Bm86 over 1:640 (Figure 4). Taken together, these findings imply that two initial doses of Gavac^plus ^at weeks 0 and 4 can induce a significant degree of tick damage suitable for the field implementation of the proposed scheme. In recent experiments, it was also demonstrated that two doses of a Bm86-based vaccine were able to affect the reproductive potential of the dog tick *R. sanguineus *due to the possible presence of cross-reactive protective epitopes in this antigen [34]. The experiment here presented provides an additional evidence for the necessity of revaccination of cattle herds if titers decline below 1:640, which tends to occur in about six months.

At present, the Gavac^plus ^vaccine is commercialized in various countries in which it is applied as part of tick control programs that combine the use of acaricide treatments with acquired immunity to Bm86. Its practical value is also preceded by more than fifteen years of research and application in the field. Although further experiments could be added to the results that are here presented, the application of the suggested scheme in the field is currently in progress.

## Conclusion

It has been demonstrated that two doses of Gavac^plus ^administered at weeks 0 and 4 to non-immunized cattle under production conditions are sufficient to elicit high anti-Bm86 specific antibodies, which are able to mediate damaging effects on ticks similar to those recorded with the use of three doses. The reduction in the number of inoculations could be a favorable issue when dealing with a large number of bovines as the stress associated with vaccination and manipulation can potentially affect milk and meat production in cattle herds.

## Authors' contributions

MV, CM, DS, DP, ER, LM, MV and AA conducted all the experiments directly related with ticks and bovines. MJ and HM determined the anti-Bm86 antibody titers by ELISA. RR and MS participated in the design of the experiments and in the analysis of results. EF carried out the statistical analyses. MPE, AR and OF participated in the analysis of the results and drafted the manuscript. All authors read and approved the final manuscript.
